# Divorcing the Late Upper Palaeolithic demographic histories of mtDNA haplogroups M1 and U6 in Africa

**DOI:** 10.1186/1471-2148-12-234

**Published:** 2012-12-03

**Authors:** Erwan Pennarun, Toomas Kivisild, Ene Metspalu, Mait Metspalu, Tuuli Reisberg, Jean-Paul Moisan, Doron M Behar, Sacha C Jones, Richard Villems

**Affiliations:** 1 Estonian Biocentre and Department of Evolutionary Biology, University of Tartu, Tartu, Estonia; 2 Division of Biological Anthropology, University of Cambridge, Cambridge, United Kingdom; 3 Molecular Medicine Laboratory, Rambam Health Care Campus, Haifa, Israel; 4 McDonald Institute for Archaeological Research, University of Cambridge, Cambridge, United Kingdom; 5 Estonian Academy of Sciences, Tallinn, Estonia

**Keywords:** mtDNA haplogroups M1 and U6, Afro-Asiatic languages, North Africa

## Abstract

**Background:**

A Southwest Asian origin and dispersal to North Africa in the Early Upper Palaeolithic era has been inferred in previous studies for mtDNA haplogroups M1 and U6. Both haplogroups have been proposed to show similar geographic patterns and shared demographic histories.

**Results:**

We report here 24 M1 and 33 U6 new complete mtDNA sequences that allow us to refine the existing phylogeny of these haplogroups. The resulting phylogenetic information was used to genotype a further 131 M1 and 91 U6 samples to determine the geographic spread of their sub-clades. No southwest Asian specific clades for M1 or U6 were discovered. U6 and M1 frequencies in North Africa, the Middle East and Europe do not follow similar patterns, and their sub-clade divisions do not appear to be compatible with their shared history reaching back to the Early Upper Palaeolithic. The Bayesian Skyline Plots testify to non-overlapping phases of expansion, and the haplogroups’ phylogenies suggest that there are U6 sub-clades that expanded earlier than those in M1. Some M1 and U6 sub-clades could be linked with certain events. For example, U6a1 and M1b, with their coalescent ages of ~20,000–22,000 years ago and earliest inferred expansion in northwest Africa, could coincide with the flourishing of the Iberomaurusian industry, whilst U6b and M1b1 appeared at the time of the Capsian culture.

**Conclusions:**

Our high-resolution phylogenetic dissection of both haplogroups and coalescent time assessments suggest that the extant main branching pattern of both haplogroups arose and diversified in the mid-later Upper Palaeolithic, with some sub-clades concomitantly with the expansion of the Iberomaurusian industry. Carriers of these maternal lineages have been later absorbed into and diversified further during the spread of Afro-Asiatic languages in North and East Africa.

## Background

The North African mitochondrial DNA (mtDNA) genetic pool has been shown to reflect influence from different regions, with sizeable portions of lineages from Sub-Saharan Africa, the Middle East, and others that diversified perhaps first in Europe [1-10], a pattern also shown with autosomal data [11]. The geographic patterns of some of the haplogroups that constitute the North African mtDNA pool have been singled out as being more informative about early population histories than others; for example, the variation in haplogroup U6 [1,12], a haplogroup that has been termed “the main indigenous North African cluster” [13], and, to a lesser extent the variation in M1, which is more predominantly found in Eastern Africa/Ethiopia [14-16]. U6 and M1 both share the feature of being African-specific sub-clades of haplogroups otherwise spread only in non-African populations. Indeed, whilst most U clades are found in North Africa and in Eurasia, as far as the Ganges Basin, U6 is virtually restricted to North (West) Africa. For macro-haplogroup M, this African connection is even more puzzling, as haplogroups belonging to M are mostly found only in South, Central and East Asia, the Americas and Oceania, where no M1 has yet been reported.

The Palaeolithic archaeological record of North Africa is spatially and temporally diverse, revealing a variety of technological shifts during the later Pleistocene period. The Aterian, a regional variant of the Middle Palaeolithic (or Middle Stone Age), was previously thought to have existed ~40,000–20,000 years ago (KYA), and argued to mark the earliest modern humans in North Africa. These dates have been drastically reassessed and the upper bound is now closer to ~115 KYA [17] or even as old as ~145 KYA [18]. The transition from the Middle Palaeolithic to Upper Palaeolithic in North Africa is characterised by the appearance of the “Dabban”, an industry that is restricted to Cyrenaica in northeast Libya and represented at the caves of Hagfet ed Dabba and Haua Fteah [19]. Whilst a techno-typological shift occurred within the Dabban ~33 KYA [19], starker changes in the archaeological record occurred throughout North Africa and Southwest Asia ~23-20 KYA, represented by the widespread appearance of backed bladelet technologies. The appearance of these backed bladelet industries more or less coincides with the timing of the Last Glacial Maximum (LGM) (~23-18 KYA), including: ~21 KYA in Upper Egypt [20]; ~20 KYA at Haua Fteah with the Oranian [21]; the Iberomaurusian expansion in the Jebel Gharbi ~20 KYA [22]; and the first Iberomaurusian at Tamar Hat in Algeria ~20 KYA [23]. The earliest Iberomaurusian sites in Morocco appear to be only slightly younger ~18 KYA [24]. Whilst backed bladelet production is broadly shared across the different regions of North and East Africa, there was also a level of regional cultural diversity during this period, possibly mirroring a diversification of populations. The Sahara Desert expanded considerably during the LGM, perhaps concentrating human groups along the North African coastal belt and the Nile Valley. Climatic conditions improved in North Africa ~15 KYA, marking the beginning of a dramatic arid-to-humid transition [25]. This increase in humidity may have opened up ecological corridors, connecting North and Sub-Saharan Africa and allowing population dispersals between the two regions. An additional arid-humid transition occurred at 11.5–11 KYA [25]; this period coincides with a widespread change in the archaeological record that marks the beginning of Capsian lithic technologies. The Capsian is argued to have developed *in situ* in North Africa, marking a continuity from the Iberomaurusian and Oranian into the Capsian [21,24,26].

Given the geographical specificity of mtDNA haplogroups U6 and M1, some studies have investigated their potential implication in the peopling of North Africa [5,27-30], whilst some earlier studies assumed that M1 diverged from other M lineages prior to the early dispersals of *Homo sapiens* out of Africa ~60–70 KYA [14,15]. However, most research that has followed explains its presence in Africa by a back-migration from Asia [5,31]. Dating of the U6 and M1 variation in African and Middle Eastern populations has been at the centre of the debate on the timing of the back-migration to Africa and, in particular, whether these haplogroups co-dispersed with certain archaeological cultures or languages. A thorough study by Olivieri and co-authors [29] proposed that both M1 and U6 were involved in an early dispersal, 40–45KYA, from Southwest Asia to North Africa in association with the first arrival of anatomically modern humans in the Mediterranean region. Considering this time frame, it was suggested, furthermore, that the spread of Aurignacian culture in Europe and the Dabban industry in North Africa derived from the same source. This interpretation was questioned by Forster and Romano who, referring to the geographic correlates, questioned this evidence and proposed that, alternatively, the spread of these haplogroups could be potentially be explained by more recent events, perhaps contemporary to the dispersal of populations speaking Afro-Asiatic (AA) languages [32].

In this study, we re-evaluate the timeframe for M1 and U6 variations and their patterns of geographic spread at the resolution of complete mtDNA sequences using a range of phylogeographic and statistical methods. We try to assess to what extent the phylogeographies of U6 and M1 are correlated with each other and, indirectly, with the spread of AA languages. In order to address these questions, a survey of more than 5700 mtDNAs was undertaken, covering a broad geographic region encompassing North and East Africa, the Near and Middle East and the Caucasus. 24 M1 and 33 U6 complete mtDNA sequences were determined and, with the refined phylogenetic trees for M1 and U6 drawn, we use this information to genotype a further 131 M1 and 91 U6 samples of different geographic origin.

## Results

### Phylogeny, phylogeography and coalescent estimates of M1 and U6

**Figure 1:**
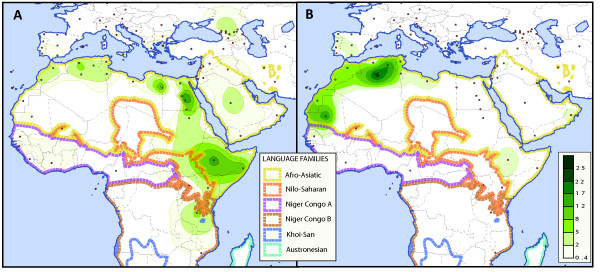
**Spatial distribution of haplogroup M1 and U6, with languages’ phyla.** Frequency maps were obtained using Surfer 8 (Golden Software, Inc.). The Kriging procedure was used and the dataset was congregated with existing ones [29] and updated with the present study, as well as the data in [27,28]. Figure 1a: frequency map for haplogroup M1. Figure 1b: frequency map for haplogroup U6. Red dots indicate the populations geographic locations.

Our genotyping of haplogroup U6 and M1 defining markers, analysed in combination with published data, confirmed earlier findings that these two haplogroups are present all over the Mediterranean Basin: both are particularly prevalent in the southern Mediterranean and M1 reaches as far away as East Africa (Figures 1a and b). Yet, some of their peak frequencies only partially overlap in Northwest Africa. In contrast to high frequencies of M1 sub-clades, haplogroup U6 is rare in East/Northeast Africa and the Middle East, and is virtually absent in the Caucasus (Table 1). Nevertheless, both haplogroups are by and large confined to the area where AA languages are spoken nowadays, being rare or absent in areas where other language families are dominant (Figure 1).

**Table 1:** Frequency of Haplogroups M1 and U6 in the geographic regions from this study

	**n**	**U6**						**M1**					
		**U6**		**U6a**		**U6b**		**M1**		**M1a**		**M1b**	
**WA**	**372**	8	2.2%	8	2.2%			4	1.1%			4	1.1%
Guinea-Bissau	372	8	2.2%	8	2.2%			4	1.1%			4	1.1%
**NWA**	**1173**	76	6.5%	56	4.8%	11	0.9%	42	3.6%	23	2.0%	19	1.6%
Morocco	530	40	7.5%	30	5.7%	4	0.8%	23	4.3%	11	2.1%	12	2.3%
Algeria	127	13	10.2%	10	7.9%	1	0.8%	2	1.6%	1	0.8%	1	0.8%
Tunisia	516	23	4.5%	16	3.1%	6	1.2%	17	3.3%	11	2.1%	6	1.2%
**NEA**	**294**	10	3.4%	10	3.4%			15	5.1%	13	4.4%	2	0.7%
Libya	101	5	5.0%	5	5.0%			1	1.0%			1	1.0%
Egypt	193	5	2.6%	5	2.6%			14	7.3%	13	6.7%	1	0.5%
**EA**	270	8	3.0%	8	3.0%			45	16.7%	45	16.7%		
Ethiopia	270	8	3.0%	8	3.0%			45	16.7%	45	16.7%		
**NE**	**1599**	13	0.8%	12	0.8%	1	0.1%	25	1.6%	22	1.4%	3	0.2%
Lebanon	171	4	2.3%	4	2.3%			2	1.2%	2	1.2%		
Saudi Arabia	205	3	1.5%	3	1.5%			8	3.9%	6	2.9%	2	1.0%
Kuwait	202							1	0.5%	1	0.5%		
Yemen	115							1	0.9%	1	0.9%		
Jordan	210	2	1.0%	2	1.0%			4	1.9%	4	1.9%		
Iran	436	1	0.2%	1	0.2%			1	0.2%			1	0.2%
Oman	80	2	2.5%	1	1.3%	1	1.3%						
Cyprus	180	1	0.6%			1	0.6%	4	2.2%	4	2.2%		
**EUR**	**1423**	5	0.4%	4	0.3%	1	0.1%	3	0.2%	3	0.2%		
Crete	193							2	1.0%	2	1.0%		
Sicily	552	4	0.7%	4	0.7%								
Russia	678							1	0.1%	1	0.1%		
**Caucasus**	**1793**	1	0.1%	1	0.1%			19	1.1%	19	1.1%		
Abkhaz	146							1	0.7%	1	0.7%		
Abazas	93							3	3.2%	3	3.2%		
Karachays	106							1	0.9%	1	0.9%		
Kumyks	112							1	0.9%	1	0.9%		
Cherkes	124							4	3.2%	4	3.2%		
Ossetians	162							4	2.5%	4	2.5%		
Kabardins	142							1	0.7%	1	0.7%		
Chechens	176							2	1.1%	2	1.1%		
Nogays	81							1	1.2%	1	1.2%		
Armenians	249	1	0.4%	1	0.4%								
Georgians	402							1	0.2%	1	0.2%		

**Figure 2:**
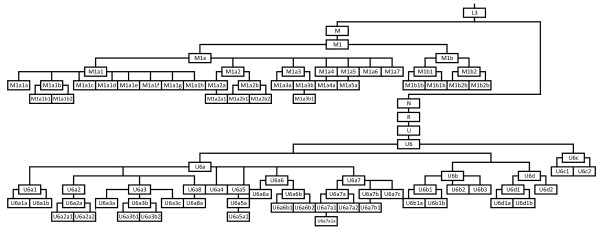
**Schematic tree of Haplogroup M1 and U6.** The tree, rooted in L3, shows the major sub-haplogroups of M1 and U6. The branching is phylogenetically correct, but the branches length is not accurate.

Concerning the estimated coalescent ages, Table 2 shows an excerpt of the Additional file 1, and contains only some coalescent ages relevant in a broader context, whilst Figure 2 shows a schematic tree of M1 and U6 phylogenies (See Additional file 2, Additional file 3, Additional file 4 and Additional file 5 for detailed phylogenies). The use of a different method (e.g. using only the synonymous mutations rather than all the mutations present in the mtDNA coding region; see [33]) for estimating molecular coalescent ages gives younger results than previously published [27-29] for both haplogroups with the coalescence of U6 at ~35 KYA and M1 at ~29 KYA. U6 is mostly prevalent in Northwest Africa (Additional file 4 and Additional file 5), a similar occurrence for M1b, which contrasts with M1a, the most diverse sub-clade of M1, for which most of its sub-clades are prevalent in East Africa. Both M1b and M1a have close coalescent ages around the LGM: ~20 and ~21 KYA respectively. M1a1 is the most diverse clade of M1a and is found in virtually all the populations where M1 has been sampled (except in Guinea-Bissau). Again, a variety of its sub-clades are more frequent in East Africa and, interestingly, a large subset of M1a1 samples could not be ascribed to any of its known sub-clades (Additional file 3). It is noteworthy to point out that all the Caucasian samples fall into just one sub-clade, M1a1b2, with no variation present at the intermediate level of resolution (Additional file 3), signature of a likely founder effect.

**Table 2:** Coalescent age estimates for M1 and U6 and some of the most frequent sub-clades

	**N**	**ρ**	**Age estimate**^**a**^	**ρ**	**Age estimate**^**b**^
M1	100	3.6	**28 900 ± 7 600**	*9.45*	*26 100*
				*95%CI*	*17 900–34 700*
M1a	78	2.62	**20 900 ± 4 900**	*7.76*	*21 200*
				*95%CI*	*15 000–27 600*
M1a1	42	2.02	**16 200 ± 3 300**	*5.43*	*14 600*
				*95%CI*	*10 400–18 800*
M1a2	15	1.93	**15 400 ± 5 700**	*6.4*	*17 300*
				*95%CI*	*9 500–25 500*
M1a3	10	1.8	**14 400 ± 6 100**	4.4	11 700
				*95%CI*	6 700–16 900
M1b	22	2.55	**20 300 ± 5 700**	7.36	20 000
				*95%CI*	12 400–28 000
M1b1	9	1.33	**10 700 ± 5 300**	3.44	9 800
				*95%CI*	3 800–14 500
M1b2	13	2	**16 000 ± 4 400**	4.62	12 300
				*95%CI*	8 000–16 800
U6	139	4.11	**32 800 ± 7 000**	12.27	34 600
				*95%CI*	24 100–45 500
U6a	104	3.74	**29 900 ± 4 600**	9.16	25 300
				*95%CI*	20 100–30 600
U6a1	22	2.73	**21 800 ± 6 400**	6.59	17 900
				95%CI	10 700–25 200
U6a2	12	1.58	**12 700 ± 4 900**	7	19 000
				95%CI	11 600–26 700
U6a3	22	2.36	**18 800 ± 5 100**	6.86	18 600
				95%CI	12 400–25 000
U6a7	27	4.7	**37 600 ± 12 800**	10.4	27 900
				*95%CI*	15 600–40 800
U6b	21	1.19	**9 500 ± 2 800**	5	13 400
				*95%CI*	7 500–19 500
U6c	5	1	**8 000 ± 5 300**	4.2	11 200
				*95%CI*	4 700–17 900

Clock: ^a^ rate from (Loogväli, et al. [33]); ^b^ calculated with the calculator from Soares et al. [34].

The most diversified sub-clade of U6 is U6a, largely due to the richness of its sub-clades in Northwest Africa. One of its sub-clades, U6a2, has been so far detected only in East African and Middle Eastern populations. Contrary to M1, various clades of U6 predate the LGM, including U6a, which is very close to the overall age of U6 (~33 KYA vs. ~36 KYA). Confirming some previous observations [27,29,30], U6b and U6c were confined in our samples to Northwest Africa.

### Bayesian Skyline Plot analyses

**Figure 3:**
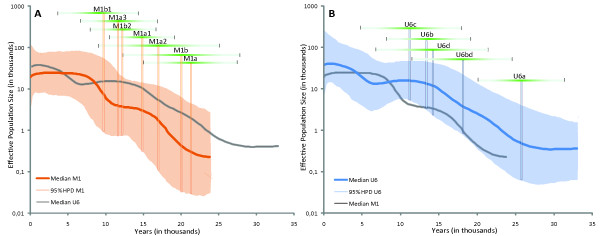
**Bayesian Skyline Plot for Haplogroups M1 and U6.** The BSPs show the variation of the Effective Population Size (Ne) through Time for M1 (Figure 3a) and U6 (Figure 3b) based on the full mitochondrial genomes. The axis scales are identical for both plots. For comparison, the median of the second haplogroup is shown in grey, but not the 95% HPD. Overlaid on the plots are the coalescent ages of some relevant sub-haplogroups, with the vertical bars indicating the calculated coalescent ages (using the calculator from [34]) and the horizontal ones their 95% confidence interval.

We tested our panel of full sequences for expansion signal(s) using Bayesian Skyline Plots (BSP) that estimate past effective population size (Ne) dynamics on the basis of sequence data [35]. The method does not rely on any pre-specified parametric model of demographic history. However, its results should be taken with caution, as the curve representing Ne could also reflect changes in the sub-structure of the population rather than its true size variation [36,37], and that the reconstruction of Ne might also be biased by the purifying selection acting on the mtDNA genome [33,34,38,39]. Yet, as here both lineages have a similar ratio of non-synonymous to synonymous mutations (0,63), this effect is not likely to explain differences that we have found. Figure 3 displays the BSPs for M1 and U6. For each simulation, the median of the other haplogroup is overlaid for comparison. We also indicate the coalescent ages and the 95% CI of some sub-clades based on the full genome as in [34], hence the coalescent ages reported in this section may differ with the ones from the previous section. The rate by Soares *et al.*[34] is applied here as the entire mtDNA genomic sequence is used for the BSP analyses, whereas the rate by Loogväli *et al.*[33] applies only to the coding region. Nonetheless, the two different approaches offer similar estimates (see Table 2 and Additional file 1 for more detail).

For U6, the initial expansion seems to more or less coincide with the ~26–27 KYA estimated coalescent age (based on full sequences) of U6a, the most diverse and prevalent sub-clade of U6. This expansion appears to have continued at a somewhat equal rate, gradually slowing down, until the curve even drops slightly, and eventually a new expansion phase takes place around ~6–7 KYA. For M1, the slope of the curve is steeper, with two clearly visible expansion phases. The first inflexion is ~22 KYA, slightly older than the estimated coalescent ages for M1a and M1b, with a strong increase until reaching a plateau at ~15 KYA. The second phase occurs at ~10–11 KYA, a time around which the estimated coalescent ages of various sub-clades of M1 fall (e.g., M1b1 and M1a1b). By directly comparing the median curves of U6 and M1, representing the past population dynamics extracted from the molecular data, it appears unlikely that the demographic histories of these haplogroups entirely overlap, both in terms of the timing of expansion phases, as well as the magnitude of these expansions.

### Mantel correlation tests

To explore whether the frequencies of M1 and U6 across a geographic range of populations correlate with languages we used Mantel correlation tests. Notably, when M1 and U6 are grouped, or with U6 alone, no significant correlation is found, neither between genes, nor geography, nor language (Table 3). A correlation is found both between geography and language only for M1, being higher with geography than with language. To see which M1 clade contributes the most to this signal, the tests were done with M1a and M1b sorted separately. No correlation could be found between M1b and geography and/or language, whilst M1a was significantly correlated both with geography and language.

**Table 3:** Mantel test to assess the correlation between genes and geography and/or language

	**Gene vs. Geography**		**Gene vs. Language**	
	**Correlation coefficient**	**p value**	**Correlation coefficient**	**p value**
M1	0.272495	0.0023	0.124358	0.0399
M1a	0.248112	0.0224	0.13911	0.0257
M1b	−0.114947	0.6141	−0.182012	0.6118
U6	0.316123	0.0576	0.244242	0.0721
M1-U6	0.10396	0.1916	0.135123	0.1002
M1/M1a	Grouped: (Abazas, Abkhaz, Cherkess, Kabardinian, Chechen, Georgia)			
	(Ethiopia,Somalia) (Greece, Crete, Cyprus) (Jordan, Lebanon, Israel) (Kuwait, Iraq)			
	(Morocco, Mauritania) (Saudi-Arabia, Yemen) (Senegal, Burkina-Fasso) (Spain, Portugal)			
	(Tunisia, Libya, Algeria)			
	Excluded: Karachays, Kumyks, Nogays, Ossetians, Kenya, Iran.			
M1b	Grouped: (Guinea-Bissau, Senegal) (Italy, Spain) (Morocco, Mauritania, Algeria)			
	(Tunisia, Libya) (Jordan, Iraq, Israel)			
U6	Grouped: (France, England, Netherlands) (Guinea-Bissau, Senegal, Nigeria)			
	(Jordan, Israel, Lebanon,Kuwait) (Morocco, Mauritania, Canarian Islands)			
	(Saudi-Arabia, Oman) (Spain, Portugal)			
	Excluded: Armenia, Ukraine, Cyprus, Iran.			
M1-U6	Grouped: (Abazas, Abkhaz, Cherkess, Kabardinian, Chechen, Georgia)			
	(Greece, Crete, Cyprus) (France, England, Netherlands) (Ethiopia,Somalia) (Spain, Portugal)			
	(Kuwait, Iraq) (Lebanon, Israel) (Morocco, Mauritania, Canarian Islands)			
	(Senegal, Burkina-Fasso, Nigeria) (Russia, Ukraine) (Saudi-Arabia, Yemen, Oman)			
	Excluded: Karachays, Kumyks, Nogays, Ossetians, Kenya, Iran, Armenia.			

The upper part of the table reports the numbers, and the lower shows the different grouping/exclusion due to sample size and/or incompatible language grouping.

## Discussion

### Origins of M1 and U6, their implications in the colonisation of North Africa, and some of its archaeological landmarks

A Southwest Asian origin has been proposed for U6 and M1 [27-29]. Yet, this claim remains speculative unless some novel “earlier” Southwest Asian-specific clades, distinct from the known haplogroups, are found in which the described so far M1 and U6 lineages are nested. Claims for basal mutations shared with M1 have recently been made in the case of haplogroup M51 and M20 (both East Asian-specific clades [40,41]): They share a root mutation (C14110T) with M1. However, one should be cautious with phylogenetic inferences drawn from these findings because this mutation is not unique in the phylogeny of mtDNA: it also occurs in the background of non-M haplogroups and therefore identity by descent within haplogroup M remains uncertain. Unfortunately, the sampling of extant populations of Africa and West Asia may not solve the question of their origin.

Assuming that M1 and U6 were introduced to Africa by a dispersal event from Asia, it would be difficult to accept their involvement in the first demographic spread of anatomically modern humans around 40–45 KYA, as suggested by Olivieri et al. (2006), [29] who associated these two clades with the spread of Dabban industry in Africa. It has indeed been previously suggested that the colonisation of North Africa from the Levant took place during the early Upper Paleolithic, as marked by the “Dabban” industry in North Africa [42]. However, comparison of early Upper Palaeolithic artefacts from Haua Fteah and Ksar Akil does not support the notion that the early Dabban of Cyrenaica is an evidence of a population migration from the Levant into North Africa [43]. Marks [44] also noted differences between the two areas in terms of the methods of blade production, further arguing against a demographic connection between the regions. Likewise, the new coalescent date estimates for M1 obtained in this study are not compatible with the model implying the spread of M1 in Africa during the Early Upper Palaeolithic, 40–45 KYA.

Given the sequence data from 242 complete sequences and genotype data of 222 mtDNAs, we were unable to find conclusive evidence that any of the geographic regions of Africa or the Middle East would stand out as being uniquely or even significantly enriched with deep-rooted clades of U6 and M1 not found elsewhere. Whilst several U6 sub-clades seem to be confined to Northwest Africa, this pattern may be the result of drift and founder effects over many thousands of years and does not necessarily suggest that Northwest Africa was the geographic source of U6 dispersals in Africa. Similarly in the case of M1b1, the Northwest African frequency pattern is apparent, whilst its counterpart, M1a, is widely spread around the Mediterranean Basin, and its current diversity is highest in East Africa. The age estimates of M1b and U6a1 (~20 KYA) together with their Northwest African-spread patterns are more consistent with their appearance during or after the spread of the Iberomaurusian culture, rather than explainable by an earlier spread of the Dabban industry. Furthermore, there is no evidence that the Dabban industry spread to NW Africa, as indicated earlier [43,44]. When taking the most recent common ancestor estimates of mtDNA haplogroups at face value and comparing them with relevant archeological horizons, then the Capsian culture also appears to be a possible candidate for the co-spread of sub-clades U6b and M1b1.

Although mtDNA is a single locus, some parallels concerning the African expansion of M1 and U6 can be drawn from autosomal data. In a recent study, Behar and colleagues explored the genome-wide diversity of the Jewish Diaspora with regard to that of their host populations, as well as the Middle East [45]. In their supplemental figure four, results of analyses undertaken with the software ADMIXTURE are shown, and specifically at K=10, an ancestry component depicted in deep purple colour appears. Interestingly, its proportion is particularly high amongst Mozabite Berbers, who have very high frequencies of M1 and U6 [12]. This deep purple colour is also present at a fairly high frequency amongst Moroccans, and to a lesser extent amongst Ethiopians, both Jewish and non-Jewish, and Egyptians. Its proportion in the Near Eastern populations is by far smaller than in the African ones.

### Mimicry of M1 and U6

A mimicry between U6 and M1 has been suggested [28,29]. Both are likely derived from a non-African ancestral clade at a similar time depth and both are largely confined to North and East Africa and the Middle East in their present-day geographic distribution. It seems, however, that the mimicry breaks down when analysing in further detail the coalescent times and frequency patterns of their sub-clades. Even at the general level, U6 is hardly found outside Northwest Africa, whilst M1 is ubiquitous throughout North Africa, East Africa and the Middle East, reaching also northern Caucasus. The coalescent age for U6a is almost 10 000 years older than that for either M1a or M1b, and most of its sub-clades coalesce before or around the LGM. In contrast, most of the estimates for M1a and M1b sub-clades are post-LGM. Also, the BSP analyses show that M1 and U6 have probably experienced different molecular histories. While the curves representing the median Ne for U6 and M1 overlap when taking the 95% HPD into account, the median curves themselves do differ. The earlier age of U6 is apparent, and though the U6 median follows a rather steady rate until declining, M1 bears testimony to two distinct expansion events. Although Hg U6 also experienced two expansion events, they do not superimpose on those of M1. It should be noted that the U6 curve should be taken with precaution as close to one half of the full U6 sequences used are from Europe. When taking into account the geography and running the BSP simulations by separate regions, it appears that the decline around 8–9 KYA is actually almost entirely driven by the European sequences (See Additional file 6). Unfortunately, it was not possible to ascertain if some of the signals present for M1 are also regional, because the number of regional sequences is too low. However, the proportion of “geographic outliers” in M1 is lower than in the case of U6.

### M1, U6 and the Afro-Asiatic language family

It has been proposed that M1 and U6, or some of their sub-clades, could be linked with the spread of AA languages [27,29,31]. Some of the main criteria for this are due to their geographical spread broadly overlapping with regions where AA languages are spoken today. There are currently two hypotheses about where AA languages originated. One places it in Northeast Africa, on the coast of the Red Sea [46,47], linking the reconstructed proto-Afro-Asiatic vocabulary to pre-Neolithic cultures in the Levant and their predecessors in southeast Egypt and northeastern Sudan, whilst the second places it in the Levant [48] , and emphasises the Neolithic component in the Afro-Asiatic cognates. Notably, even the earliest time frame (~10 KYA or more) considered by the linguists [47,49] for the earliest splits in the language family are more recent than the ages of U6 or M1 and their major sub-clades. However, if the sub-clades of M1 and U6 were to be involved in the dispersal event associated with the Afro-Asiatic languages they had to exist at the moment of the launch of this event, and therefore the fact that these sub-clades are older makes them plausible candidates for such dispersal. However, when considering M1 and U6 as a whole, or U6 alone, no correlation with language (and geography) was found with the current data, indicating for U6 that its expansion was not concomitant with that of the AA.

Concerning haplogroup M1 individually, a significant correlation with languages was observed. Furthermore, within M1, it appears that the correlation is mostly due to M1a. However, given the small sample size of M1b, any potential signal correlating with language might not be detectable. Interestingly, M1a has a likely East African origin, but its coalescent age of ~21 KYA still largely predates that of the proto-AA. Maybe a sub-clade of M1a would still give a similar correlation, but there are not sufficient samples to allow splitting M1a into its various sub-clades, and to test for a correlation. Although we found a correlation, limited sample sizes do not allow drawing unambiguous connection between genes and languages. Furthermore, it is also possible that this putative sub-clade of M1 does not testify for the expansion of AA speaking people, but was already present among the people who inhabited the area before the spread of the AA languages.

## Conclusions

Our analyses do not support the model according to which mtDNA haplogroups M1 and U6 represent an early dispersal event of anatomically modern humans at around 40–45 KYA in association with the spread of Dabban industry in North Africa as proposed earlier [28,29]. A West Asian origin for these haplogroups still remains a viable hypothesis as sister clades of U (and ancestral to it, macro-hg N (including R)) and M are spread overwhelmingly outside Africa, notably in Eurasia, even though the phylogeographic data on extant populations do not present a clear support for it. Our estimates of coalescent times and demographic analyses of U6 and M1 variations suggest that their spread in North and East Africa is largely due to a number of demographic events, predominantly occurring at the end of the peak of as well as after the LGM, but largely before the Holocene. Hence, some of the topologically earliest sub-clades of U6 and M1 may have been involved in the origin and spread of the essentially North African Iberomaurusian culture, and the observed correlations with languages make it likely that the North and East African carriers of the two matrilineages have been absorbed into the expanding Afro-Asiatic languages-speaking people in the area, but in phylogeographically differential ways.

## Methods

### Samples

From over 5700 samples spanning Europe and countries around the Mediterranean Basin and beyond, 153 M1 and 121 U6 samples were identified based on their HVSI variation and then confirmed by RFLP (all unrelated individuals, who gave their informed consent). Samples from the literature/GenBank were retrieved, including: 77 M1 (2 from [50], 1 from [51], 3 from [52], 8 from [28], 1 from [53], 2 from [39], 1 from [54], 2 from [55], 2 from [56], 51 from [29], 1 from [57], 3 from [58] and 3 from [59]); and 93 U6 (1 from [60], 6 samples from Family Tree DNA deposited in GenBank, 1 from [61], 2 from [53], 1 from [62], 12 from [27], 30 from [29], 2 from [57], 39 from [30] and 7 from [58]). 9 samples were corrected (See Additional file 7 for the corrected positions) compared to their current GenBank entry at the time of this article’s submission, including 2 from [28], 1 from [55] and 5 from [27] (Dr. Vicente Cabrera’s personal communication). Also, 2 M1 and 3 U6 samples were kindly provided by Family Tree DNA (with some U6 samples having a potential match to sequences deposited in GenBank, see Additional file 7 and its legend for more details), bringing the total to 236 and 230 samples for M1 and U6 respectively (See Additional file 7 for detailed information). All the work complied with the Helsinki Declaration of Ethical Principles (59^th^ WMA General Assembly, Seoul October 2008). The Estonian Basic Research project SF0182474 was approved by the Research Ethics Committee of the Estonian Biocentre.

### Sequencing, SNP typing

The 153 M1 samples from this study have been screened for approximately 2 kb of coding region in 4 separate fragments (between nps 700–1080, 6250–6990, 12590–13146, 14750–15580) chosen to cover some SNP-defining sub-clades of M1 based on previous knowledge [16,55]. 22 samples were fully sequenced following previously published protocol [63], and slightly modified. Based on the tree drawn from 105 full (or nearly full) sequences (Additional file 2), some SNPs have been typed in order to place precisely all the samples on the tree (See Additional file 3 and Additional file 8 for the full typing information).

For the 121 U6 samples, several fragments have been amplified to type SNPs of interest based on the samples’ HVS I information (See Additional file 9 for full typing information), as well as from the tree based on 139 full sequences (See Additional file 4).

### Phylogenic tree, network

The trees and network were drawn by hand and checked with Network 4.5.1.0 ( http://www.fluxus-technology.com/[64]). If needed, a weighing scheme was used for highly recurrent polymorphisms.

### Coalescent age estimates

For the coalescent age calculations, the rho (ρ) statistic and standard deviation were used as in [65,66] but see [67] for a critical assessment of it. Different rates were used: For the coding region, rate [33] is used, and for the full genome, estimates were calculated with the calculator provided in [34]. For all calculations, 2 M1 samples from [50] and the 3 M1 from [52] were discarded – the first ones were missing several portions of the coding region, and the second ones seemed to exhibit potential sequence errors (See [68] for details). For the full genome calculations, further samples were discarded, as their control region is not reported (M1: 1 from [53] and 2 from [39]; U6: 2 from [53], and 1 from [62]).

### Mantel test

The haplotype of each sample was composed of all the polymorphisms detected in the coding region during the genotyping of each haplogroup, with the missing polymorphisms assumed to be similar to the RSRS [58], plus the control region (16024–16400). The HVS II was excluded of the haplotype, as it was only sequenced in some samples and, unlike for the coding region, it cannot be reasonably assumed that a specific polymorphism is absent in a different sample. For some populations the sample size was small, in which case they were grouped with a close geographic neighbour sharing the same language family. If this was not possible, the samples/populations were excluded (See Table 3 for details). The genetic distance matrices were based on Slatkin’s linearised FSTs. Because the language families present in the data are too divergent to rank and order them, we used a binary approach, with populations (or grouped populations) speaking a language from the same language family given a distance of 0, and a distance of 1 otherwise. Mantel tests were done with Arlequin 3.5.1.2 [69], with 100,000 permutations.

### Bayesian Skyline Plot

The Bayesian Skyline Plot (BSP) [35] is a graphical depiction of the variation of the effective population size (Ne) through time. BSP analyses were performed with the software BEAST v1.5.4 [70]. The GTR substitution model was used with a gamma distribution, plus invariant sites. An uncorrelated lognormal relaxed clock was applied [71]. The whole mitochondrial genome was used, and runs were performed for 40,000,000 generations with 20 groups. In order to assure that convergence was reached, several independent runs were done (See Additional files 10 and Additional file 11 for M1 and U6, respectively). Also, the impact of the number of groups, which was user defined, was explored (See Additional file 12 and Additional file 13 for M1 and U6, respectively) by increments of 5, from 5 to 50. The axes were converted into their final units (effective population size vs. time) with a rate of 1,695 × 10^-8^[34] and a generation time of 25 years. But in order to take into account the purifying pressure acting on the whole molecule, ρ was deduced from the data, and then entered into the calculator provided in [34], resulting in a time scale which can be put in comparison with the coalescent ages calculated in the same way. Accordingly, the samples which were not available in full, with some missing parts, or which might suffer from errors (See the paragraph on coalescent age estimates) were not included in the analyses. Additional file 14 and Additional file 15 show the differences when the overall rate vs. the rate taking into account purifying selection [34] are used for plotting the results; the major impact being upon the time axis, whereas the impact on the effective population size or the overall shape of the curve are minimal.

## Competing interests

The authors declare that they have no competing interests.

## Authors’ contributions

RV, TK and JPM conceived the study, EP and DMB performed the full sequencing, EP, EM and TR genotyped the samples, EP performed the statistical analyses. EP, TK and RV interpreted the results and drafted the manuscript, MM designed some figures, SCJ revised the manuscript focusing on the archeological aspect. All authors read and approved the final manuscript.

## Supplementary Material

Additional file 1 **– Coalescent age estimates for M1 and U6 and their most frequent sub-clades.** Soares^a^: These estimates include some sequences that are not complete, and are given just for indication, see the left panel for estimates based only on complete sequences. Click here for file

Additional file 2 **– Phylogenetic tree based on 105 M1 full sequences.** All positions are scored against the RSRS [58] and are transitions, unless followed by a capital letter that marks the resulting transversion. Indels are scored with i or d, heteroplasmies follow the IUB code and reversal to ancestral state by an exclamation mark (!), double back mutations by two exclamation marks (!!). The positions are colour coded according to their status: purple – non-coding; blue – non-synonymous; and black – synonymous. Variations in the C tracts were mostly ignored (i.e., 16182C, 16193C, 309+2C, etc.) unless stated on the tree. The box containing the sample ID is colour coded according to the publications from which they were retrieved (See the main text for the full reference), and below it their geographic origin is colour coded (See Additional file 7 for the specifics). Sequences available only for the coding region, or for which some parts are missing, are flagged with a yellow mark under the geographic origin. The order for the root mutation(s) for M1a1g, M1a1h, M1a7 and M1b2c were deduced from additional partial sequencing (See Additional file 3). Click here for file

Additional file 3 **– Network based on 236 M1 samples.** All positions are scored against the RSRS [58] and are transitions, unless followed by a capital letter that marks the resulting transversion. Indels are scored with i or d, heteroplasmies follow the IUB code and reversal to ancestral state by an exclamation mark (!), double back mutations by two exclamation marks (!!). The positions are colour coded according to their status: purple – non-coding; blue – non-synonymous; and black – synonymous. Variations in the C tracts were ignored (i.e., 16182C, 16193C, 309+2C, etc.). The box containing the sample ID is colour coded according to the publications which they are from (See the main text for the full reference), and below it their geographic origin is colour coded (See Additional file 7 for the specifics). Click here for file

Additional file 4 **– Phylogenetic tree based on 139 U6 full sequences.** All positions are scored against the RSRS [58] and are transitions, unless followed by a capital letter that marks the resulting transversion. Indels are scored with i or d, heteroplasmies follow the IUB code and reversal to ancestral state by an exclamation mark (!). The positions are colour coded according to their status: purple – non-coding; blue – non-synonymous; and black – synonymous. Variations in the C tracts were mostly ignored (i.e., 16182C, 16193C, 309+2C, etc.) unless stated on the tree. The box containing the sample ID is colour coded according to the publications which they are from (See the main text for the full reference), and below it their geographic origin is colour coded (See Additional file 7 for the specifics). Sequences available only for the coding region are flagged with a yellow mark under the geographic origin. The potential reticulation created by position 150 between sub-clades U6a3a and U6a3c was resolved on the more frequent occurrence of 150 in various different haplogroups’ backgrounds (See [72]). We refined here the phylogeny of the Canary-specific branch formerly known as U6b1 [27,29]. There is an array of 2 common mutations before the branch splits into the so-called Canary-specific branch and one apparently specific to Northwest Africa. We propose therefore to rename U6b1a as U6b1a1 to comply with the revised phylogeny. The mutations order of some clades (U6a1a1b, U6a1a2, U6a2b, U6a2b1, U6a3d, U6a3d1, U6a3d1a, U6a6a, U6b1b1) was deduced for additional partial typing (see Additional file 5). Click here for file

Additional file 5 **– Network based on 230 U6 samples.** All positions are scored against the RSRS [58] and are transitions, unless followed by a capital letter that marks the resulting transversion. Indels are scored with i or d, heteroplasmies follow the IUB code and reversal to ancestral state by an exclamation mark (!). The positions are colour coded according to their status: purple – non-coding; blue – non-synonymous; and black – synonymous. Variations in the C tracts were ignored (i.e., 16182C, 16193C, 309+2C, etc.). The box containing the sample ID is colour coded according to the publications which they are from (See the main text for the full reference), and below it their geographic origin is colour coded (See Additional file 7 for the specifics). The reticulation created by position 150 in U6a3’s clades is left unresolved. Click here for file

Additional file 6 **– BSP for U6 based on North African and European sequences separately.** For the North African and European sequences, only a few independent runs were done to ascertain that convergence was reached. The 10 convergence runs for all sets of sequences are shown for comparison. Click here for file

Additional file 7 **– List of the 466 samples.** GeoBroad abbreviations are as follow: WA: West Africa; EA: East Africa; EUR: Europe; NE: Near/Middle East; NWA: North-West Africa; NEA: North-East Africa. See the main text for the full references. In the case of 4 samples originally provided by Familly Tree DNA, 3 samples have an identical sequence that matches an entry in GenBank, and as they cannot be differentiated, they have not been separately deposited into GenBank. For the last sample, there are two entries in GenBank with an identical sequence, and thus that sample as well has not been deposited into GenBank. Click here for file

Additional file 8 – Genotyping information for 153 M1 samples. Click here for file

Additional file 9 – Genotyping information for 121 U6 samples. Click here for file

Additional file 10 **– 10 independent BSP runs for M1 with 20 groups.** All runs were performed using the same parameters. Click here for file

Additional file 11 **– 10 independent BSP run analyses for U6 with 20 groups.** All runs were performed using the same parameters. Click here for file

Additional file 12 – BSP for M1 with groups varying from 5 to 50 groups, in increments of 5. Click here for file

Additional file 13 – BSP for U6 with groups varying from 5 to 50 groups, in increments of 5. Click here for file

Additional file 14 **– BSP for M1 with the corrected rate versus uncorrected.** The uncorrected rate use a rate of 1,695 x 10^-8^[34], and the corrected rate was deduced with the deduced rho values from the time, using the calculator from [34]. Click here for file

Additional file 15 **– BSP for U6 with the corrected rate versus uncorrected.** The uncorrected rate use a rate of 1,695 × 10^-8^[34], and the corrected rate was deduced with the deduced rho values from the time, using the calculator from [34]. Click here for file
