# Supplementary figures and images for: Divorcing the Late Upper Palaeolithic demographic histories of mtDNA haplogroups M1 and U6 in Africa
Source: BMC Evol Biol. 2012 Dec 3;12:234. doi: 10.1186/1471-2148-12-234 (PMC3582464; doi:10.1186/1471-2148-12-234)

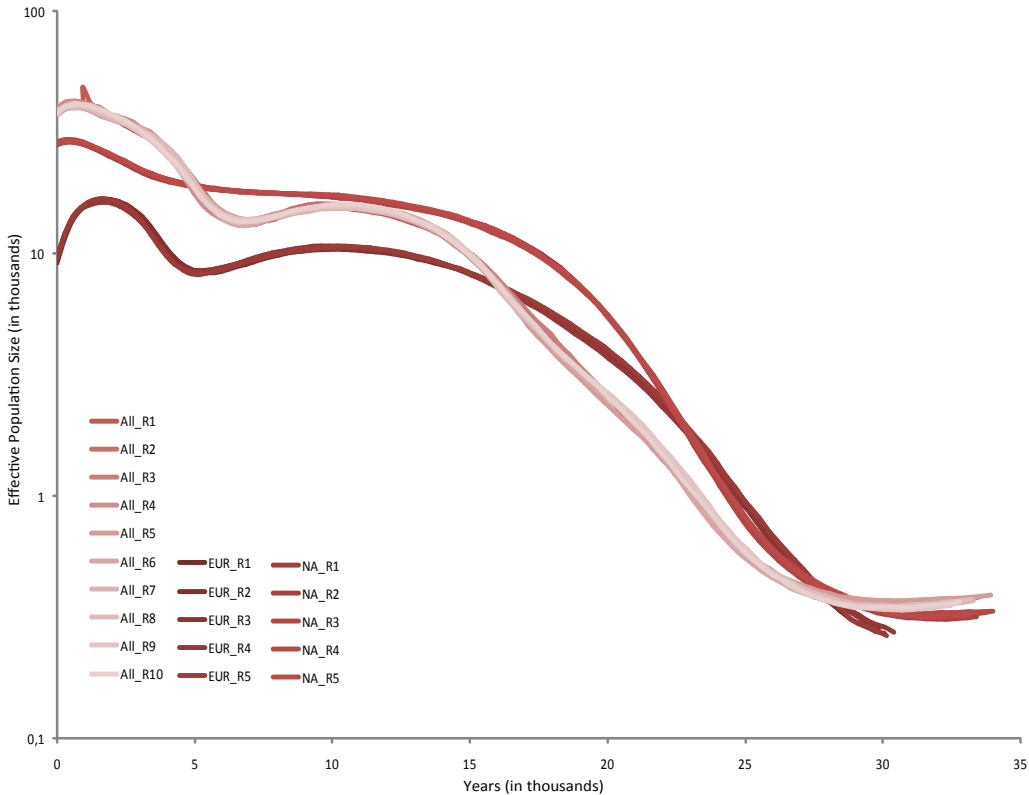

Supplement: Additional file 6 — – BSP for U6 based on North African and European sequences separately. For the North African and European sequences, only a few independent runs were done to ascertain that convergence was reached. The 10 convergence runs for all sets of sequences are shown for comparison. [file 1471-2148-12-234-S6.pdf]

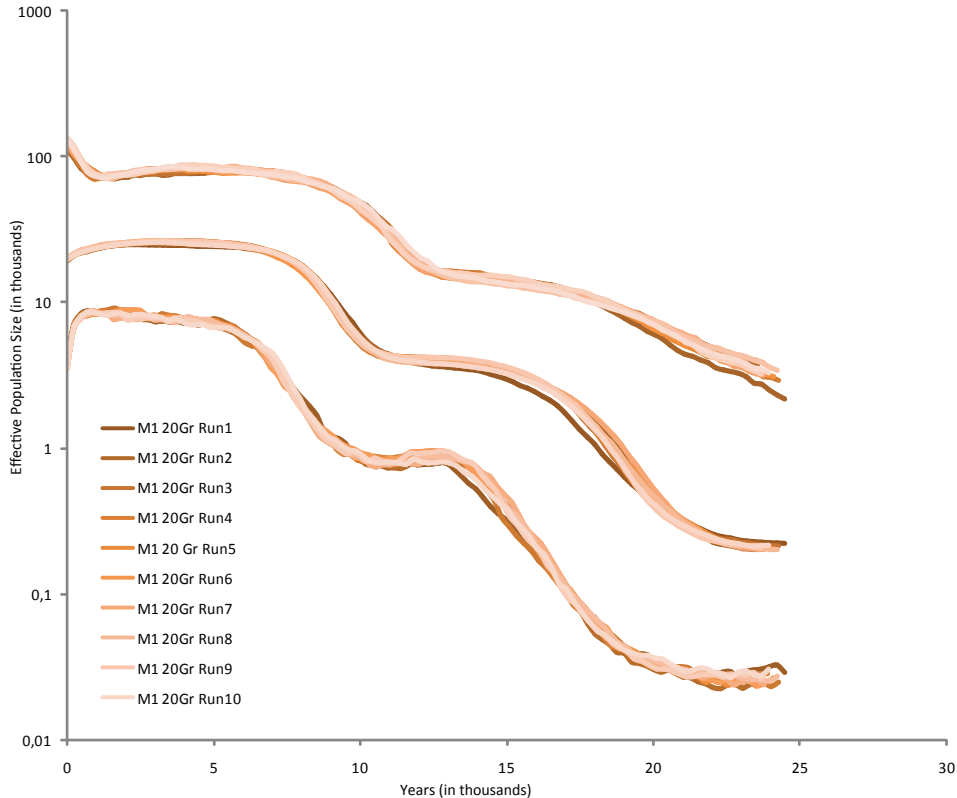

Supplement: Additional file 10 — – 10 independent BSP runs for M1 with 20 groups. All runs were performed using the same parameters. [file 1471-2148-12-234-S10.pdf]

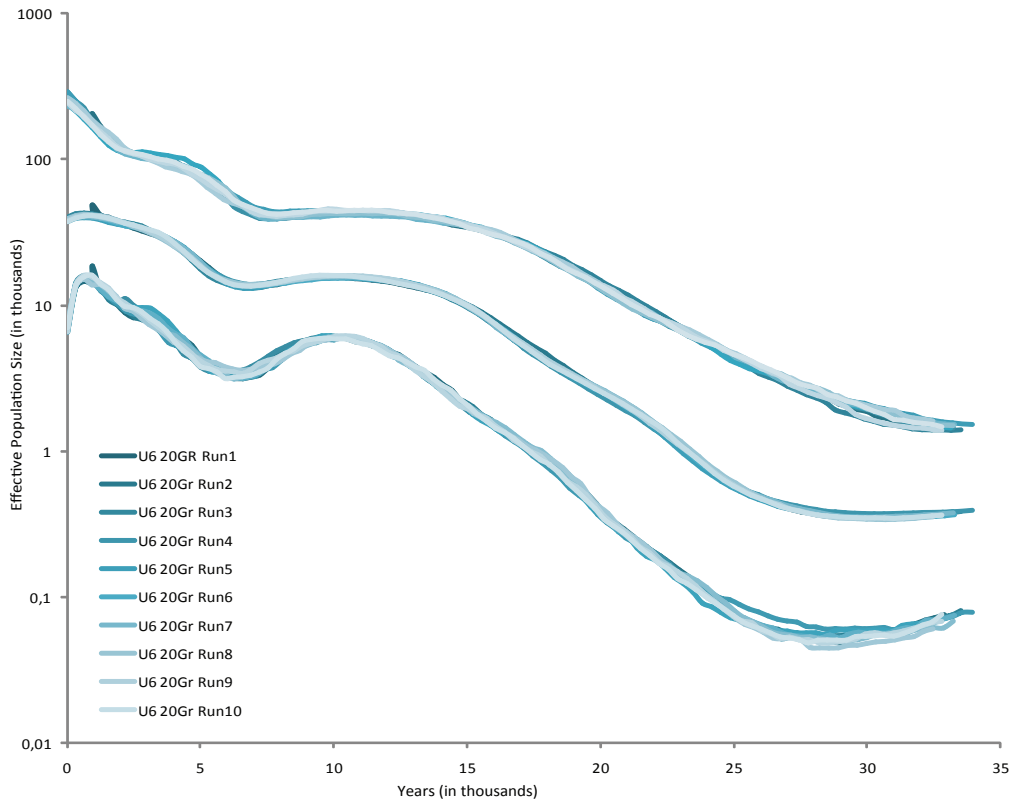

Supplement: Additional file 11 — – 10 independent BSP run analyses for U6 with 20 groups. All runs were performed using the same parameters. [file 1471-2148-12-234-S11.pdf]

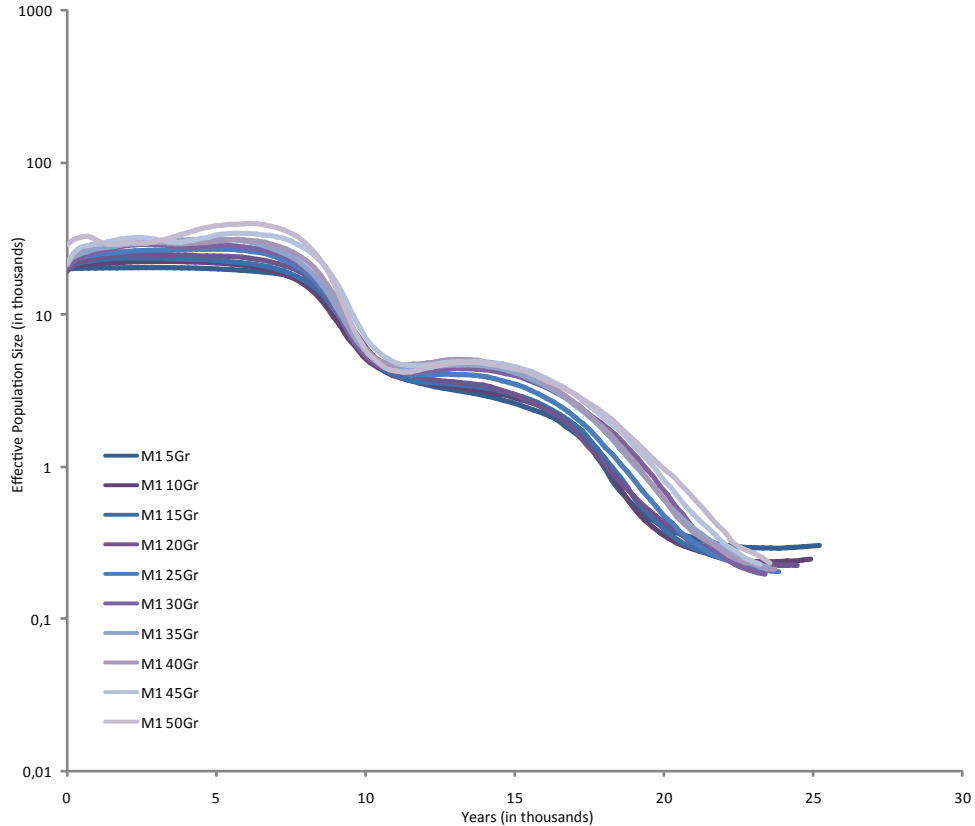

Supplement: Additional file 12 — – BSP for M1 with groups varying from 5 to 50 groups, in increments of 5. [file 1471-2148-12-234-S12.pdf]

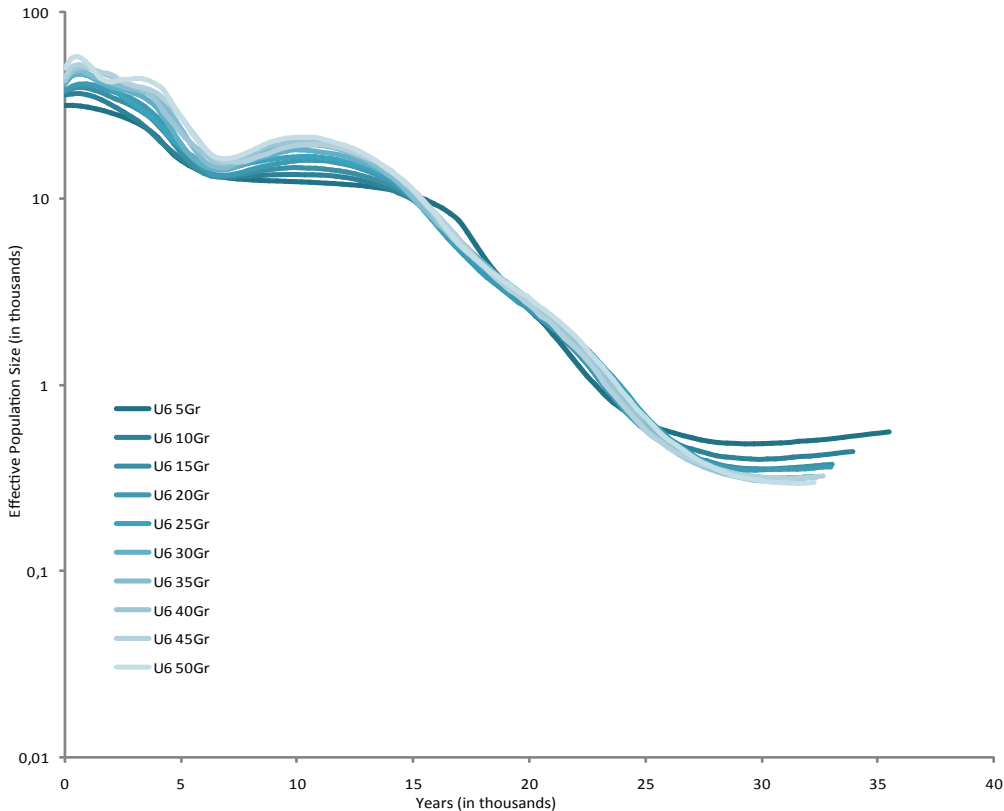

Supplement: Additional file 13 — – BSP for U6 with groups varying from 5 to 50 groups, in increments of 5. [file 1471-2148-12-234-S13.pdf]

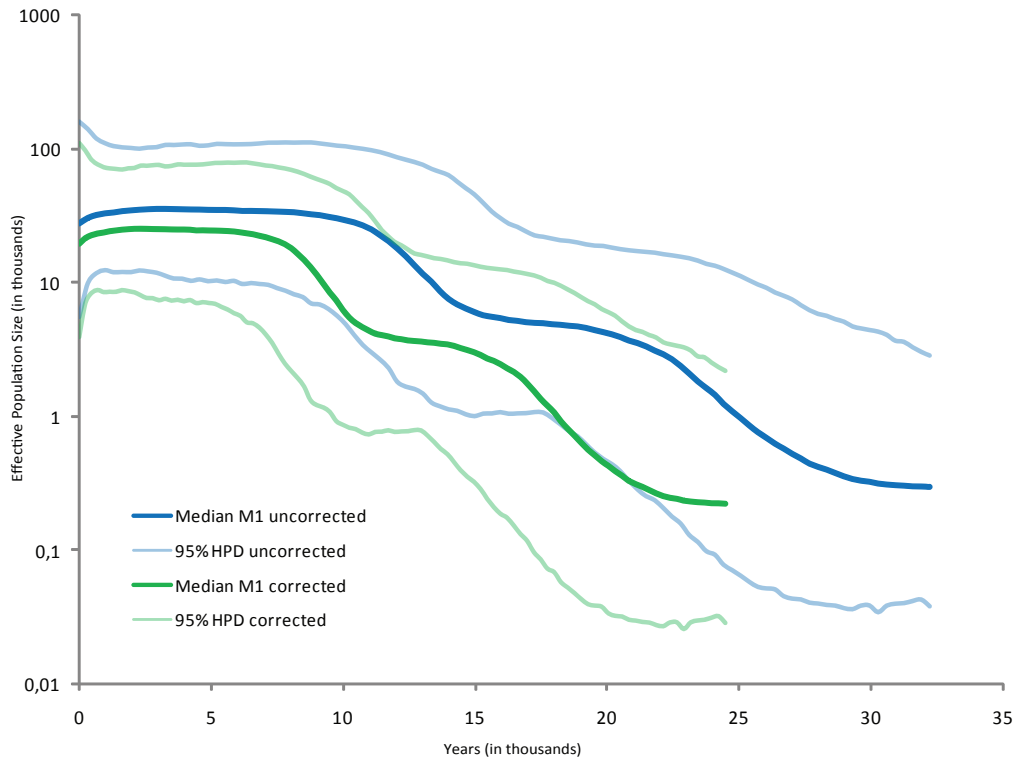

Supplement: Additional file 14 — – BSP for M1 with the corrected rate versus uncorrected. The uncorrected rate use a rate of 1,695 x 10^-8^[34], and the corrected rate was deduced with the deduced rho values from the time, using the calculator from [34]. [file 1471-2148-12-234-S14.pdf]

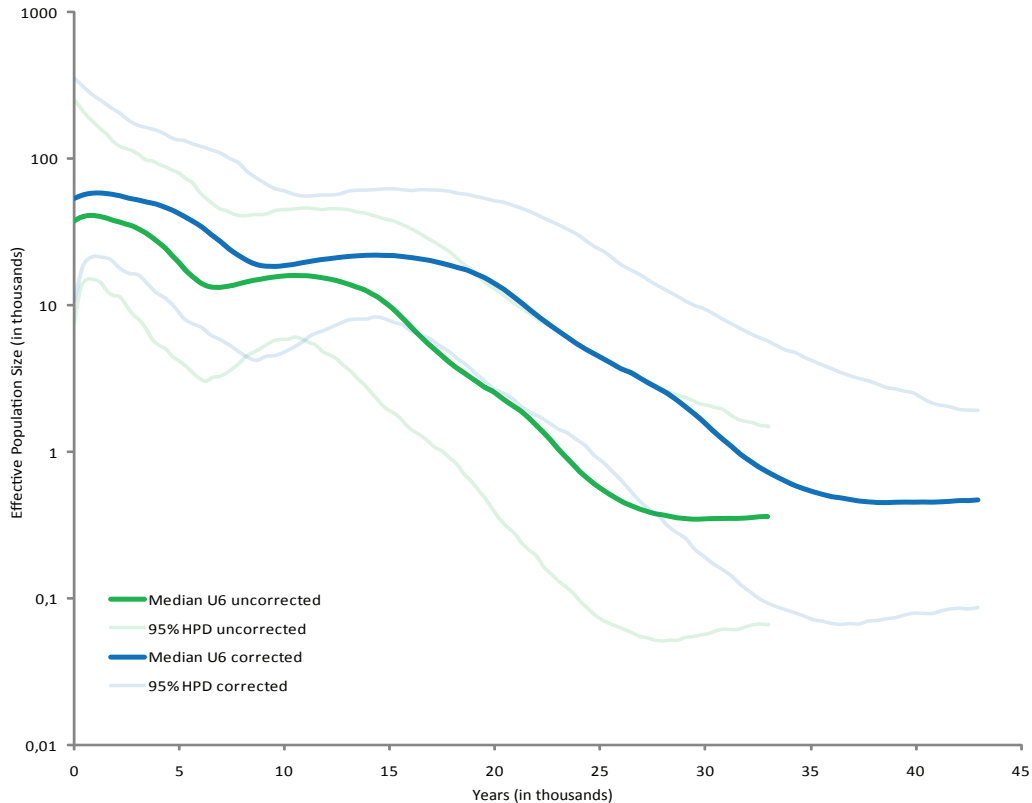

Supplement: Additional file 15 — – BSP for U6 with the corrected rate versus uncorrected. The uncorrected rate use a rate of 1,695 × 10^-8^[34], and the corrected rate was deduced with the deduced rho values from the time, using the calculator from [34]. [file 1471-2148-12-234-S15.pdf]
